# Role of Leptin in Obesity, Cardiovascular Disease, and Type 2 Diabetes

**DOI:** 10.3390/ijms25042338

**Published:** 2024-02-16

**Authors:** Teresa Vilariño-García, María L. Polonio-González, Antonio Pérez-Pérez, Josep Ribalta, Francisco Arrieta, Manuel Aguilar, Juan C. Obaya, José A. Gimeno-Orna, Pedro Iglesias, Jorge Navarro, Santiago Durán, Juan Pedro-Botet, Víctor Sánchez-Margalet

**Affiliations:** 1 Department of Medical Biochemistry and Molecular Biology, and Immunology, School of Medicine, Virgen del Rocio University Hospital, University of Seville, Seville 41013, Spain; tvgarcia@gmail.com; 2 Department of Medical Biochemistry and Molecular Biology, and Immunology, School of Medicine, Virgen Macarena University Hospital, University of Seville, 41009, Spain; maria.polonio@hotmail.com (M.L.P.-G.); antonioresi@gmail.com (A.P.-P.); 3 Departament de Medicina i Cirurgia, University Rovira i Vigili, IISPV, CIBERDEM, 43007 Tarragona, Spain; josep.ribalta@urv.cat; 4 Endocrinology and Nutrition Service, Ramón y Cajal University Hospital, 28034 Madrid, Spain; arri68@hotmail.com; 5 Endocrinology and Nutrition Service, Puerta del Mar University Hospital, Instituto de Investigación e Innovación en Ciencias Biomédicas de la Provincia de Cádiz (INiBICA), Cádiz University (UCA), 11001 Cádiz, Spain; manuelaguilardiosdado@gmail.com; 6 Chopera Helath Center, Alcobendas Primary Care,Alcobendas 28100 Madrid, Spain; juancarlosobaya@yahoo.es; 7 Endocrinology and Nutrition Department, Hospital Clinico Universitario Lozano Blesa, 15 50009 Zaragoza, Spain; jagimeno@salud.aragon.es; 8 Endocrinology and Nutrition Service, Puerta de Hierro University Hospital, Majadahonda, 28220 Madrid, Spain; piglo65@gmail.com; 9 Hospital Clínico Universitario de Valencia,46011 Valencia, Spain; jorgenavper@gmail.com; 10 Endodiabesidad Clínica Durán & Asociados,41018 Seville, Spain; sduran@duransanz.com; 11 Lipids and Cardiovascular Risk Unit, Hospital del Mar, Autonomous University of Barcelona, 08003 Barcelona, Spain; jpedrobotet@psmar.cat; 12 Institute of Biomedicine of Seville (IBIS), Hospital Universitario Virgen del Rocío/Virgen Macarena, CSIC, Universidad de Sevilla, 41013 Seville, Spain

**Keywords:** type 2 diabetes mellitus, obesity, leptin, cardiovascular risk, inflammation

## Abstract

Diabetes mellitus (DM) is a highly prevalent disease worldwide, estimated to affect 1 in every 11 adults; among them, 90–95% of cases are type 2 diabetes mellitus. This is partly attributed to the surge in the prevalence of obesity, which has reached epidemic proportions since 2008. In these patients, cardiovascular (CV) risk stands as the primary cause of morbidity and mortality, placing a substantial burden on healthcare systems due to the potential for macrovascular and microvascular complications. In this context, leptin, an adipocyte-derived hormone, plays a fundamental role. This hormone is essential for regulating the cellular metabolism and energy balance, controlling inflammatory responses, and maintaining CV system homeostasis. Thus, leptin resistance not only contributes to weight gain but may also lead to increased cardiac inflammation, greater fibrosis, hypertension, and impairment of the cardiac metabolism. Understanding the relationship between leptin resistance and CV risk in obese individuals with type 2 DM (T2DM) could improve the management and prevention of this complication. Therefore, in this narrative review, we will discuss the evidence linking leptin with the presence, severity, and/or prognosis of obesity and T2DM regarding CV disease, aiming to shed light on the potential implications for better management and preventive strategies.

## 1. Introduction

Diabetes mellitus (DM) has evolved into a mounting epidemic over the past century, with a more conspicuous escalation in recent decades driven by the exponential surge in obesity. DM has emerged as one of the foremost global causes of mortality. Presently, it ranks as the seventh leading cause of death in the United States, with 400,000 deaths per year and 5.2 million deaths worldwide [1]. Type 2 DM (T2DM) arises from inadequate insulin production by the pancreas and insulin resistance [2,3]. The increase in DM prevalence is closely linked to the rise in overweight and obesity conditions. In 2022, the World Heart Federation revealed that 2300 million adults are affected by overweight/obesity, with expected increases even in low- and middle-income countries, primarily due to nutritional transition, i.e., the shift from traditional diets to westernized diets [4]. Obesity is involved not only in the etiopathogenesis of the most common global DM, T2DM, but also in the development of its complications. However, an increasing number of studies are exploring its role in T1DM as well [5].

Regarding complications in these patients, studies demonstrate that individuals with T2DM carry an elevated risk of cardiovascular disease (CVD) mortality across various ethnic and gender groups compared to those free from this disease. This mortality risk is further exacerbated when obesity coexists. Obesity contributes to 44% of deaths in individuals with T1DM and 52% of deaths in T2DM cases. Common CVD manifestations among people with DM include heart failure (HF), peripheral arterial disease (PAD), and coronary artery disease (CHD) [1,6].

The pathogeneses of DM and obesity share common pathways involving insulin resistance, oxidative stress, and prothrombotic and proinflammatory patterns. In an obese milieu, where overfeeding is stimulated, a disruption in the metabolic equilibrium occurs, resulting in the subsequent deposition of fat in organs not specialized in lipid storage (termed ectopic fat), such as the endothelium, liver, and skeletal muscle. This gives rise to metabolic alterations and disorders, including insulin resistance, atherosclerosis, and T2DM, culminating in cardiovascular, cerebrovascular, and hepatic diseases. Moreover, obesity leads to heightened leptin levels and inflammatory markers such as C-reactive protein (CRP), further exacerbating vascular and myocardial injuries [7].

Leptin is a protein classified within the adipokine family and is currently recognized as a hormone consisting of 167 amino acids with a molecular weight of 16 kD. It is predominantly synthesized in adipocytes, and its production is contingent upon the volume of fat deposits in the body [8,9]. Leptin traverses the blood–brain barrier (BBB) to exert its central action and interact with hypothalamic receptors situated in neurons across various nuclei, including the arcuate, paraventricular, and centromedial ones. The primary site of action is the arcuate nucleus within the hypothalamus, comprising two distinct populations of neurons. One releases neuropeptide Y, constituting the orexigenic pathway (appetite inducer), while the other secretes proopiomelanocortin, forming the anorexigenic pathway (satiety inducer). Numerous studies affirm that leptin inhibits the orexigenic pathway and stimulates the anorexigenic pathway, underscoring its pivotal role in body weight regulation [10,11,12].

As mentioned earlier, leptin is a hormone capable of effectively reducing food intake and body weight, initially suggesting its potential in obesity treatment. However, it has been observed that obese individuals exhibit high levels of circulating leptin and insensitivity to exogenous leptin administration. The inability of leptin to exert its anorexigenic effects in obese individuals and, consequently, the lack of clinical utility in obesity are defined as leptin resistance. Understanding the molecular mechanisms underlying leptin resistance is crucial for the effective application of leptin in obesity treatment. One key aspect is the necessity for leptin to cross the BBB to reach the hypothalamus and exert its anorexigenic functions. Several mechanisms within this context are not fully understood, and, in recent years, new strategies have been developed to restore the response to leptin in patients with obesity [12].

Elevated levels of leptin may function as an indicator of leptin resistance. Mechanisms contributing to leptin resistance include genetic mutations, autoregulation of leptin, restricted tissue access, and molecular/cellular circulatory regulation [13]. The evidence indicates that central leptin resistance is a key factor in the development of obesity. Concurrently, the resultant leptin resistance associated with obesity can adversely affect various peripheral tissues, including the liver, pancreas, platelets, vasculature, and myocardium. This metabolic and inflammatory damage may arise from either resistance to leptin action in specific tissues or an excess of leptin action attributed to obesity-related hyperleptinemia.

Several studies suggest that leptin deficiency poses a notable risk for obesity and CVD. Leptin resistance represents a complex and not fully understood pathophysiological phenomenon, emphasizing the need for further investigation due to its clinical implications [12,14,15]. Numerous research findings have established a connection between leptin, its resistance, and diseases like DM and obesity. These conditions often lead to severe complications, such as CVD, making it crucial to conduct comprehensive studies in this area. A deeper understanding of these associations can guide the development of preventive measures for at-risk individuals and potential therapeutic interventions. To contribute to this knowledge, we have reviewed the literature to underscore the potential role of leptin in cardiovascular disease among obese patients with T2DM.

## 2. Obesity and CVD

The prevalence of obesity has surged uncontrollably since the 1960s, as evidenced by long-term follow-up surveys, including the Behavioral Risk Factor Surveillance System, the National Health Interview Survey, and the National Health and Nutrition Examination Survey (NHANES). This increase has reached epidemic proportions since 2008, solidifying its status as one of the paramount global health challenges.

The prevalent parameter for assessing overweight and obesity conditions is the body mass index (BMI). Individuals with a BMI ≥ 25 kg/m^2^ are categorized as overweight, while those with a BMI ≥ 30 kg/m^2^ are classified as obese. However, the BMI scale has notable limitations, such as its inability to account for variations in body composition. Athletes with a higher lean body mass may be overestimated, whereas the elderly with reduced lean mass may be underestimated. Alternative methods for evaluating body composition include waist circumference, waist-to-hip ratio, height, skinfold thickness, and dual-energy X-ray absorptiometry. Waist circumference, specifically, serves as a straightforward and reliable measure for assessing abdominal obesity—an established CVD risk factor independent of BMI. This parameter is particularly valuable for individuals classified as normo- or overweight [16].

Obesity, recognized as a chronic metabolic disease, exerts a substantial impact on the cardiovascular system [17]. This influence stems from various mechanisms, including hemodynamic alterations, modifications in cardiac structure and function, inflammation, neurohumoral shifts, and cellular remodeling. Collectively, these factors contribute to a reduction in cardiac output, heightened peripheral resistance, augmented left ventricular (LV) mass, thickening of the LV wall, enlargement of internal dimensions, and compromised systolic function of the left ventricle [6,7,18].

Obesity can lead to structural remodeling of the heart, resulting in LV hypertrophy. For every 1 kg/m^2^ increase in BMI, the risk of LV hypertrophy increases by 5.1%, and, for every 1 cm increase in waist circumference, the risk of LV hypertrophy increases by 2.6% [19].

Conversely, the enlargement of adipocytes may directly correlate with a phenotypic shift from M2 to M1 macrophages, triggering the production of leptin, angiotensin, proinflammatory cytokines (such as IL-1, TNF-α, and IL-6, etc.), and reactive oxygen species [20]. Moreover, the progression of inflammation, oxidative stress, and heightened leptin levels in obesity is strongly correlated with the development of CVD and hypertension [18,21,22]. Leptin demonstrates pleiotropic effects. In obese individuals, hyperleptinemia is insufficient for preventing the dysregulation of energy balance, suggesting that individuals with obesity may exhibit leptin resistance. 

While most cases of obesity are associated with hypothalamic leptin resistance, the peripheral effects of leptin signaling or leptin resistance in obesity are not fully elucidated. Moreover, the net effects of hyperleptinemia or leptin resistance on CVD in obese individuals are complex and still under investigation. Obesity has been recognized as an independent predictor of CHD, influencing it indirectly through its effects on related comorbidities such as dyslipidemia, hypertension, glucose intolerance, endothelial dysfunction, and inflammation. It is associated with increased arterial stiffness, coronary artery calcification, thickening of the carotid intima-media, and a higher incidence of carotid stenosis, all contributing to premature vascular aging. This leads to an approximately twofold-higher risk of developing heart failure than a subject with a healthy weight. Studies have also shown correlations among stroke and BMI and waist-to-hip ratios. Population-based cohort studies have reported a 49% higher risk of atherosclerosis in obese patients compared to non-obese individuals. This is attributed to the release of proinflammatory adipokines by visceral adipose tissue, increased oxidative stress from heightened free radical production by adipose tissue, impaired autophagy, endothelial dysfunction, and activation of adipose tissue macrophages, T cells, and B cells within fat deposits, all contributing to plaque formation and fibrous lesions [6,7,18].

On the other hand, an obesity paradox emerged from various cross-sectional studies in patients with heart failure, in which a higher survival rate was observed in those who were overweight or obese (BMI > 27.8 kg/m^2^). Consequently, a follow-up analysis was conducted in patients with chronic heart failure, revealing significantly lower mortality rates in those patients with an elevated BMI. This trend was also observed in cases of sudden cardiac arrest, coronary artery disease, and diabetes. Generally, obese subjects exhibit higher mortality rates; however, paradoxically, a higher mortality is often found in normal-weight patients compared to obese patients. This phenomenon, where survival rates are higher among obese patients, contrary to conventional expectations, is referred to as the “obesity paradox”, and it is most frequently observed in patients with CHD or HF. A possible explanation may be that people with obesity typically also present with an increased amount of lean mass, which is associated with improved cardiorespiratory fitness [23]. The increase in LM may explain part of the obesity paradox as it is associated with improved cardiorespiratory fitness. Nevertheless, the paradox may reflect an epidemiological artifact rather than a true negative association between a normal weight and clinical outcomes [7,18].

Moreover, the World Health Organization defines metabolically healthy obesity as individuals with grade II obesity or higher who do not have DM. Other authors characterize it as obese individuals who do not have DM, hypertension, and/or hyperlipidemia. The definition of metabolically healthy obesity is not well-established, and there is ongoing debate regarding whether it constitutes a genuine entity or a transient phase in the progression of overall obesity [24]. Furthermore, obesity has recently been found to be an independent risk factor of myocardial fibrosis regardless of metabolic health [25].

Furthermore, the precise contribution of central and visceral obesity to the onset of cardiovascular disease (CVD) is still not fully understood. In a study involving one million men examining the impact of cardiorespiratory fitness and obesity during adolescence on subsequent chronic disability from CVD [26], it was observed that being overweight or obese was linked to disability across all the examined causes of CVD. These findings imply associations between low levels of cardiorespiratory fitness and obesity and the subsequent risk of chronic disability arising from CVD [27].

## 3. Obesity and Diabetes

Overweight and obesity are intricate conditions closely linked to T2DM [5,28]. Recently acknowledged as a disease by the World Health Organization (WHO), obesity, but not overweight, is considered a primary catalyst for insulin resistance. This resistance manifests in the liver, white adipose tissue, and skeletal muscle. Consequently, pancreatic beta cells respond by inadequately secreting insulin in an attempt to overcome this resistance, a phenomenon observed in the early stages of the disease [28,29].

Given the escalating prevalence of type 2 diabetes mellitus (T2DM), researchers have primarily associated it with the increasing rates of obesity, peripheral insulin resistance, minority ethnic groups, and a family history of T2DM. The World Health Organization’s 2002 STEPS survey revealed a 21.5% prevalence of T2DM and a 54.8% prevalence of obesity. Subsequent surveys in 2013 showed a surge in T2DM prevalence to 45.8%. Projections for 2030 estimate that 552 million people worldwide could be affected by T2DM. In a meta-analysis comparing obese individuals to those with a normal weight conducted in the United States and Europe, obese men exhibited a 7-fold-higher risk, while obese women had a 12-fold-higher likelihood of developing T2DM [1,5].

Moreover, additional studies have demonstrated that the distribution of adipose tissue is a critical independent factor, irrespective of the degree of obesity, in the development of insulin resistance [30]. The authors conducted a comparison between a group of obese adolescents with a similar adiposity index. Adolescents with an impaired glucose tolerance (IGT) exhibited greater insulin resistance compared to those with a normal glucose tolerance [31]. Moreover, individuals with an impaired glucose tolerance exhibited elevated intramyocellular lipid content and increased visceral fat deposition, while experiencing a reduction in subcutaneous fat deposition. Ebe D’Adamo et al. [32] discovered that children with a high proportion of visceral fat and limited abdominal subcutaneous fat demonstrate higher insulin resistance and elevated plasma glucose levels during the second hour of the glucose tolerance test. Additionally, it has been reported that ectopic hepatic fat deposition in obese patients is a significant marker of insulin resistance and glucose dysregulation [5].

However, there are several additional risk factors for DM aside from obesity [5]. Thus, insulin resistance is associated with various conditions, including acanthosis nigricans, early puberty, hypertension, dyslipidemia, and polycystic ovary syndrome.

Ethnicity also plays a role in the predisposition to type 2 diabetes mellitus (T2DM). The SEARCH study (Type 2 Diabetes Mellitus in Pediatric Age) found a higher predisposition in Native American children, followed by Asian, African American, Hispanic, and, lastly, non-Hispanic white children [33] 

Family history is a significant factor. In a study conducted by Almiri et al. [34], over half of the patients with prediabetes and all those with T2DM had a first-degree relative with T2DM. The participants with prediabetes (20%) and DM (70%) also had elevated triglyceride levels.

A recent study conducted in Abu Dhabi [35] involving 216 obese (90%) or overweight (7%) patients found that children with greater height, weight, and waist circumference for their age exhibited higher insulin resistance. Among this group, 7% already had diabetes, 8.2% had impaired glucose tolerance (IGT), and 18.1% had impaired fasting glucose.

Adipose tissue serves as an energy depot, controlling lipid mobilization and playing a crucial role in energy regulation and glucose homeostasis. It comprises preadipocytes/adipocytes-like cells, macrophages, endothelial cells, fibroblasts, and leukocytes. Additionally, adipose tissue functions as an endocrine organ by secreting adipokines, such as leptin, adiponectin, resistin, visfatin, apelin, vaspin, hepcidin, chemerin, and omentin. These adipokines exert metabolic effects in muscle, liver, pancreas, and the brain [36].

Recently, studies have highlighted the significance of obesity-induced inflammation in predisposing individuals to develop DM, with this inflammation being mediated by immune cells in local tissues. It is noteworthy that the inflammation triggered by obesity shares many characteristics with inflammation induced by infection, involving both the innate and adaptive immune systems [37,38]. The inflammatory status in obesity may, at least in part, result from the activation of the cells of the immune system by leptin [13,39,40], including monocytes, T lymphocytes, and natural killer (NK) cells [41].

The relationship between the immune system, NK cells, obesity, and DM has been previously studied [42]. Thus, depleting NK cells appears to improve insulin resistance in individuals with a high percentage of body fat. However, in those patients without a high-fat diet, NK cell depletion has been shown to have no impact on metabolic outcomes. Consistent with this hypothesis, the opposite approach, expanding NK cells with IL-15, worsens insulin resistance in those subjects with high-fat diets. It has also been observed that, as obesity increases, adipose tissue macrophages are activated, and they have two primary actions [42]. First, they produce chemokines that attract circulating NK cells, thereby increasing their number in epididymal fat; and second, they synthesize IL-15, activating and expanding NK cells in epididymal fat, further promoting the increase in adipose tissue macrophages (cross-activation). This cycle that is established induces inflammation in obesity and, consequently, insulin resistance through the actions of various mediators in the liver and muscle that release epididymal fat [42]. In this context, recent data also support the contribution of NK cells of visceral adipose tissue in the low-grade inflammation associated with insulin resistance [43].

## 4. Diabetes and CVD

In recent decades, DM has steadily increased and has become one of the leading global causes of death [1]. CVD also poses a significant burden on public health, representing the leading causes of death worldwide. CVD is responsible for approximately 17.9 million deaths annually, constituting 31% of all deaths globally. Consequently, cardiovascular prevention, early detection, and management of established CVD risk factors such as dyslipidemia, T2DM, and hypertension are crucial components of public health policy [44].

Among patients with T2DM, CVD stands as the leading cause of mortality, contributing to 52% of deaths [44,45]. This outcome is influenced significantly by both genetic and metabolic components. Furthermore, microvascular complications, such as nephropathy, retinopathy, and neuropathy, also play a role in mortality. The prevalent manifestations in these patients encompass heart failure (HF) and peripheral arterial disease (PAD), with coronary heart disease (CHD) following these as a prominent macrovascular disease [46,47].

Consequently, individuals with type 2 diabetes mellitus (T2DM) face an elevated risk of stroke. Within the National Health and Nutrition Examination Survey (NHANES) cohort, 26.3% of strokes were linked to DM, presenting a twofold-higher risk for ischemic stroke and a 50% increase for hemorrhagic stroke. Additionally, these patients experience heightened mortality rates following acute myocardial infarction and hypertension [46,47].

A majority of individuals with T2DM exhibit elevated blood pressure levels. Hypertension and DM are well-established risk factors for CVD. The presence of both conditions further amplifies this risk compared to individuals with either condition alone. This heightened risk is thought to result from a synergistic effect on both large and small blood vessels simultaneously, limiting the potential for compensatory collateralization which protects organs from the adverse consequences of vascular damage. The primary function of vasculature is to deliver oxygen and nutrients to tissues, such as the heart, brain, or kidneys. Functional changes occurring in T2DM and hypertensive conditions significantly alter hemodynamic stress on the heart and other organs, leading to these consequences [46]. Patients with T2DM commonly exhibit increased arterial stiffness and dysfunction of vascular endothelial cells. Coexistence of hypertension exacerbates this phenomenon. Several mechanisms have been proposed to explain this pathophysiology. Elevated blood glucose is a significant determinant of both arterial stiffness and carotid intima-media thickness, with the latter being a well-established measure of blood pressure-related damage which independently predicts CVD events. Chronic hyperglycemia is associated with the accumulation of advanced glycation end products, contributing to arteriosclerosis. This could elucidate the impact of blood glucose on endothelial function. Additionally, hyperglycemia is associated with oxidative stress, inflammation, the formation of advanced glycation end products, abnormalities in calcium homeostasis, and apoptosis. These factors contribute to the structural remodeling of the heart [48]. A meta-analysis indicated that patients with T2DM with an increase in carotid intima-media thickness of 0.13 mm are associated with a nearly 40% higher risk of cardiovascular disease compared to control subjects [46].

Certain cardiovascular disease (CVD) risk factors, including smoking, hypertension, hypercholesterolemia, or metabolic syndrome, independently contribute to CVD in patients with T2DM. In a study involving 371,221 veterans, it was observed that two-thirds of diagnosed patients with T2DM also had comorbid dyslipidemia and hypertension, conditions whose prevalence was doubled in patients with T2DM compared to those without T2DM [49]. The presence of metabolic syndrome traits in T2DM further increases the risk of CVD in individuals with chronically elevated blood glucose and impaired plasma glucose homeostasis. Given that excessive visceral fat and ectopic fat are the primary drivers of metabolic syndrome, special attention is recommended for the prevalent subgroup of patients with T2DM who are also viscerally obese and exhibit metabolic syndrome components, as they are considered a high-risk phenotype for adverse CVD outcomes [50]. Moreover, there are studies indicating that inflammatory markers such as CRP in T2DM can provide prognostic information. Elevated levels of CRP may imply a higher risk of CVD and PAD in these patients [1].

Regarding gender differences in T2DM, specific variations have been highlighted in the Early Rancho Bernardo study [51]. The study demonstrated that men with T2DM had a 2.4-times-higher risk of ischemic heart disease compared to men without it, while women had a 3.5-times-higher risk, along with higher myocardial infarction mortality, and experienced earlier occurrence [1].

Intensive glycemic control interventions have demonstrated a reduction in the risk of macrovascular complications compared to less intensive strategies. Regarding metformin, there is controversy surrounding its ability to prevent cardiovascular events, as some studies have failed to establish a clear relationship. However, findings from the SPREAD-DIMCAD trial, which included 304 patients with T2DM and coronary artery disease, confirmed the CVD-protective effect of metformin [52]. Patients treated with metformin exhibited a 46% risk reduction (*p* = 0.026) in recurrent CVD events compared to patients treated with sulfonylurea glipizide [44].

Recently, it has been confirmed that new antidiabetic drugs, such as SGLT2 inhibitors and GLP1 antagonists, are effective in reducing CVD consequences, independent of their impact on glucose levels [53]. Moreover, initiating positive lifestyle changes such as regular exercise, smoking cessation, and adopting a healthy diet at the onset of glucose abnormalities could be beneficial in preventing the development or progression of the disease and reducing the incidence of CVD [45]. In addition to antidiabetic agents, statins and new drugs that lower LDL levels such as PCSK-9 inhibitors have been reported to improve endothelial dysfunction, contribute to the regression of vascular plaque, and enhance microvascular reactivity in patients with T2DM and dyslipidemia. These findings suggest positive outcomes in terms of CVD morbidity and mortality for this class of drugs [54].

## 5. Leptin

Leptin, a 16 kDa protein encoded by the obesity gene (ob), was discovered in 1994 and plays a crucial role in the regulation of weight and energy homeostasis [8,55]. Despite its name, derived from the Greek word “leptos”, meaning thin, higher circulating levels of leptin have been observed in obese individuals compared to those with a normal weight [56]. It functions as a proinflammatory adipokine, contributing to the “low-grade inflammatory state” often seen in overweight and obese patients. Leptin is a key player in the field of immunometabolism [39]. 

Produced predominantly by subcutaneous white adipose tissue (WAT) rather than visceral adipose tissue [57,58], leptin expression is under the influence of various hormones, including insulin [59] and glucocorticoids [60,61], with an inverse circadian rhythm. Leptin exhibits autocrine, paracrine, and endocrine signaling capabilities to distant tissues [62,63,64]. Its secretion follows a circadian pattern, peaking at night and decreasing during the day. Women generally have higher leptin levels than men with the same weight, attributed to their higher percentage of lean body mass [65].

Leptin exerts its effects on food intake and energy expenditure by binding to and activating the long form of its membrane receptor (LEPR-B), which is prominently expressed in the hypothalamus and other brain regions, resulting in appetite suppression. For this action, leptin needs to traverse the blood–brain barrier (BBB). As a result, leptin is classified as an “anorexigenic” hormone, promoting a reduction in appetite.

However, in obese individuals, there is often a condition of hyperleptinemia due to leptin resistance, and, conversely, where leptin resistance is present, it can contribute to elevated leptin levels [66,67]. This intricate interplay highlights the complexities associated with the role of leptin in obesity and its impact on metabolic regulation.

Leptin is implicated in CVD events, and hyperleptinemia has been linked to the presence and severity of coronary heart disease (CHD) and HF [18,68,69,70]. Additionally, leptin plays a significant role in reproductive processes [18,71,72,73,74]. The diverse roles of leptin in both cardiovascular health and reproduction underscore its multifaceted impact on various physiological functions within the body.

Various studies have shown that circulating leptin concentrations decrease during fasting or energy restriction but increase during overfeeding as well as during surgical stress. These findings highlight the dynamic regulation of the leptin signaling system by different pathways to maintain body mass [12].

The leptin receptor LepR is expressed in various tissues, including adipose tissue, heart, muscle, lung, small intestine, liver, and the central nervous system, particularly the hypothalamus [75]. This widespread expression of leptin receptors facilitates the pleiotropic effects of leptin. Typically, leptin is released from adipocytes into the bloodstream, crosses the blood–brain barrier, and reaches specific areas of the brain, particularly the hypothalamus, which is involved in the regulation of energy balance [11].

LepR belongs to the type I cytokine receptor family, and t has six subtypes obtained through splicing (ranging from LepRa to LepRf) [76]. Following LepR autophosphorylation, leptin signaling takes place, triggering the Janus kinase (JAK) tyrosine kinase pathways and the signal transducer and activator of transcription (STAT), insulin receptor substrate (IRS)/phosphatidylinositol 3-kinase (PI3K), mitogen-activated protein kinase (MAPK), extracellular signal-regulated kinase (ERK), and adenosine 5′-monophosphate-activated protein kinase (AMPK) pathways [77,78]. The activation of each of these pathways contributes to the anorexigenic effects of leptin (appetite suppression, weight loss stimulation, and increased thermogenesis) [79,80,81].

Insulin serves as the primary regulator of leptin production. Prolonged elevation in insulin levels leads to an increase in leptin concentration, although this effect has only been demonstrated with prolonged insulin elevation [12].

LepRa can activate MAPK, IRS1, and PI3K [82,83], but LepRb (long isoform, also known as OB-R) is the only one capable of mediating intracellular JAK/STAT signaling, a very important pathway in energy homeostasis [84]. After LepR binds to leptin, LepR undergoes a conformational change, crucial for leptin signaling and the activation of JAK2, which autophosphorylates and phosphorylates tyrosine residues in the intracellular domain of functional LepR, allowing the binding of STAT proteins and their subsequent tyrosine phosphorylation, dimerization, and translocation to the nucleus, where they act as transcription factors (forming a dimer which enters the nucleus to regulate the expression of genes which govern food intake) [77,85]. The suppressor of cytokine signaling 3 (SOCS3) and protein tyrosine phosphatase 1B (PTP1B) can negatively regulate the JAK/STAT pathway, suppressing it, leading to negative feedback within this signaling pathway [86,87].

Another signaling pathway of the leptin receptor involves MAPK or ERK. Leptin binding results in the phosphorylation of protein tyrosine phosphatase 2 (SHP2) containing SH2, and growth factor receptor-bound protein 2 (Grb2) activates ERK. Likewise, JAK2 activates signaling through Grb2 and SHP2. LepR activation facilitates interaction and the formation of JAK2/IRS1 complexes to regulate protein kinase B (Akt) from PI3K, which is a dimer and has a component which regulates this signaling by acting as a catalyst [83,88].

**Figure 1:**
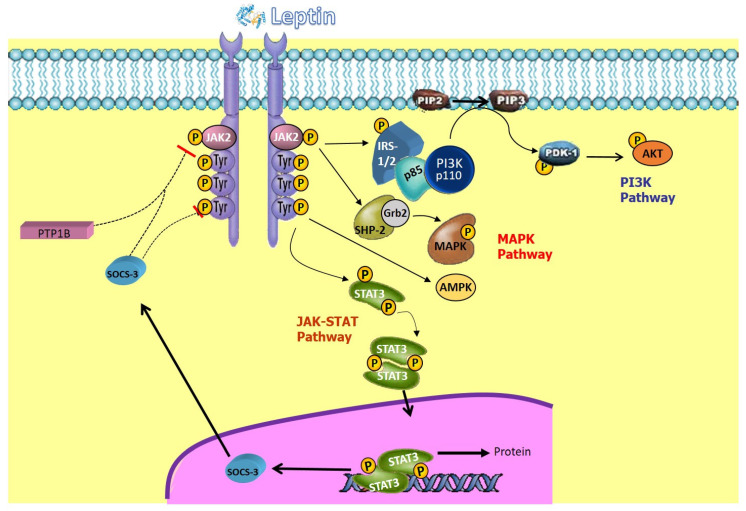
Signal transduction of leptin receptor. Leptin signaling is based on the autophosphorylation of the leptin receptor, triggering the JAK2-STAT3, IRS/PI3K, MAPK-ERK, and AMPK pathways. JAK, Janus kinase; IRS, insulin receptor substrate; PI3K, phosphatidylinositol 3-kinase; PDE, phosphodiesterase; Akt, protein kinase B; SHP2, tyrosine-protein phosphatase 2 containing SH2; Grb2, growth factor receptor-bound protein 2; MAPK, mitogen-activated protein kinase; PTP, tyrosine-protein phosphatase; STAT, signal transducer and activator of transcription; ERK, extracellular signal-regulated kinase; SOCS3, cytokine signaling 3 suppressor; AMPK, 5′-adenosine monophosphate-activated protein kinase.

The JAK2/STAT3 pathway is primarily responsible for regulating changes in gene expression, while the PI3K pathway plays a significant role in the acute effects of leptin, producing rapid cellular responses by promoting changes in ion channels and, therefore, cellular activity, such as the regulation of food intake and blood pressure. The JAK/STAT3, MAPK, and PI3K pathways via SH2-B collectively appear to regulate energy balance [76,89] (Figure 1).

The regulation of AMPK by the leptin receptor is tissue-specific [90]. Thus, in the hypothalamus, leptin inhibits AMPK to reduce appetite [91], whereas leptin stimulates AMPK activity in skeletal muscle, increasing fatty acid oxidation [92]. In fact, AMPK is pivotal in energy homeostasis and in the majority of diseases associated with metabolic syndrome, such as diabetes, obesity, hypertension, etc., and this is why AMPK-activating drugs reverse many of the metabolic defects associated with insulin resistance [93].

In the hypothalamus, leptin interacts with a receptor, suppressing hunger and stimulating satiety. Some of these interactions involve neuropeptide Y (a hunger promoter) or proopiomelanocortin (POMC) neurons by binding to the same type of receptors. Here, these neurons are also targeted by insulin; thus, the actions of leptin and insulin are interconnected and contribute to desirable metabolic control. Insulin is responsible for maintaining proper energy storage and utilization, while leptin reduces the ongoing energy intake. Therefore, both are necessary for the central regulation of energy expenditure and glucose homeostasis [94]. Additionally, leptin enhances the synthesis of the alpha-melanocyte-stimulating hormone (α-MSH), which suppresses long-term appetite. Additionally, it can influence short-term hunger suppression through cholecystokinin [95,96,97].

Leptin acts as an acute-phase reactant, increasing the secretion of proinflammatory cytokines such as IL-6, IL-12, and TNF-α in macrophages. This, in turn, increases leptin expression in adipose tissue and circulating leptin, creating a feedback loop which promotes the inflammatory state [13,41]. Obese individuals exhibit a greater abundance of macrophages in their adipose tissue, indicating that both adipocytes and macrophages play significant roles in inflammation in obesity. This is attributed to the secretion of interleukin-1 (IL-1) and tumor necrosis factor alpha (TNF-α), establishing an additional feedback loop. This association elucidates the connection between obesity and chronic proinflammatory signaling pathways, abnormal cytokine production, and increased acute-phase reactants as well as the increased risk of developing inflammatory diseases and disorders mediated by the immune system [39].

## 6. Leptin and Obesity

The absence of leptin or its receptors results in uncontrolled hunger, potentially leading to obesity. Numerous studies have demonstrated a strong correlation between leptin levels and body fat percentage, with an increase in adipocytes triggering elevated leptin as an adaptive response for energy balance control [56,98]. Patients with leptin deficiency have been observed to develop obesity and hyperphagia in childhood, prompting the consideration of leptin replacement therapy to suppress appetite and increase energy expenditure. However, many obese patients persist in their condition even with elevated leptin levels, suggesting the possibility of leptin resistance. This resistance may contribute to an increased calorie intake and hinder sustained weight loss [99,100].

The concept of “leptin resistance” emerged shortly after the discovery of leptin in 1994 [8]. This resistance stems from obesity-related processes that impair leptin function, hindering its ability to reach target cells. This impairment can result from reduced expression of leptin receptors (LepR) or altered signaling pathways, ultimately contributing to the progression of obesity. Despite elevated leptin levels, hypothalamic neurons may become less sensitive to leptin or fail to respond appropriately. Consequently, this resistance diminishes the effectiveness of exogenous leptin therapy in obese individuals [12,100,101]. Leptin resistance may arise from various factors, including alterations in leptin expression, mutations in leptin or its receptors, or disruptions in leptin transport across the blood–brain barrier (BBB) [101,102,103,104,105]. Although the precise molecular mechanisms underlying decreased leptin transport remain unclear, it is hypothesized that there could be saturation and degradation of the transporters (BBB receptors) due to excess leptin or reversible inhibition induced by external factors [103].

A specific subtype known as selective leptin resistance has been identified, characterized by the absence of leptin’s effects on appetite and body mass regulation. However, the sympathetic nervous system remains responsive to leptin in this subtype. Leptin, in this context, continues to exert its influence on the sympathetic nervous system, promoting lipid mobilization in white adipose tissue and inducing thermogenesis in brown adipose tissue through the increased expression of uncoupling protein 1 (UCP1) [101]. Two potential theories have been proposed to explain selective leptin resistance. The first theory suggests a defect in the molecular signaling pathways that specifically mediate leptin’s actions in certain locations rather than universally. The second theory posits defects in specific brain regions that regulate the specific actions of leptin. Moreover, recent research implicates the brain renin-angiotensin system (RAS) as a mediator of renin’s effects on the thermogenic renal autonomic nervous system, with no observed effects of leptin on food intake in this context [104].

In the context of obesity, alterations in sexual dimorphism are observed in response to leptin. For instance, obese males exhibit a maintained or increased sympathetic-excitatory response, while obese females experience a limited sympathetic-excitatory response to leptin, leading to disruptions in the reproductive cycle. These changes are likely associated with alterations in sexual dimorphism in neuropeptide Y (NPY) and proopiomelanocortin (POMC) participants in the paraventricular nucleus of the hypothalamus. However, the precise mechanisms of leptin action in obese women and men are not entirely clear [106].

## 7. Leptin and T2DM

Elevated leptin levels are associated with insulin resistance and type 2 diabetes mellitus (T2DM). Within this specific population, there is an association between hyperleptinemia and a higher risk of CVD as well as a higher percentage of obesity, hypertension, and endothelial dysfunction [106]. Leptin plays a role in regulating proinflammatory cytokines, which are also associated with insulin resistance and T2DM, including the tumor necrosis factor and IL-6. Neurons expressing proopiomelanocortin (POMC) in the arcuate nucleus of the hypothalamus are involved in the effects of leptin on glucose homeostasis in obese and insulin-resistant patients. These neurons activate anorexigenic neurons that express POMC and cocaine- and amphetamine-regulated transcript (CART) while inhibiting orexigenic neurons expressing neuropeptide Y (NPY) and the agouti-related peptide (AgRP) [106,107].

During fasting, a reduction in serum leptin levels stimulates the expression of AgRP and NPY while suppressing POMC and CART. This physiological response leads to an increased food intake and reduced energy expenditure [108].

Experimental studies in T2DM suggested an improvement in insulin resistance and a suppression of hepatic gluconeogenesis and fasting hyperglycemia in animals receiving leptin replacement therapy [109].

There is evidence that sitagliptin (dipeptidyl peptidase-4 inhibitor) can reduce leptin concentrations in both humans and animals, as can liraglutide (a glucagon-like peptide-1 receptor agonist) [110]. Metformin is also capable of reducing leptinemia and has demonstrated an increase in leptin receptor expression in the liver of mice, along with an improvement in the hypothalamic sensitivity to leptin [111].

As a general concept, hyperleptinemia has been linked to the presence of insulin resistance, T2DM, and macroangiopathy. It is worth noting that certain antidiabetic drugs, including metformin, pioglitazone, sitagliptin, liraglutide, and empagliflozin, have been shown to reduce leptin levels, although the clinical implications of these drugs’ effect have not yet been fully elucidated [110,112].

## 8. Leptin and CVD

In animal models of obesity with mutations in the ob gene or LepR, heart failure was commonly observed [113,114]. This suggests a connection between leptin and CVD [115]. In most cases, hyperleptinemia is associated with unfavorable outcomes in CV disorders. In fact, leptin may be produced by the heart to function as an autocrine/paracrine factor [116]. Nevertheless, documented cases in rodent studies suggest that leptin-deficient or LepR-deficient animals may exhibit a protective effect on the heart. This protection is attributed to coronary artery vasodilation, the activation of endothelial nitric oxide synthase (eNOS), and the activation of endothelial precursor cells. These studies also concluded that leptin levels are higher in women compared to men [18,70,117]. 

In the Framingham study involving 818 participants (average age 79 years, 62% women), leptin levels were strongly associated with congestive HF and CVD. After adjusting for BMI, the association with congestive HF was abolished, but the relationship with a higher incidence of CVD development was only modestly attenuated [68,118].

The expansion in blood volume in obese individuals results in a heightened cardiac output, triggering stress and structural remodeling. This, in turn, can lead to cardiac hypertrophy. Studies have shown that administering a leptin antagonist locally can mitigate angiotensin II-induced ascending aortic aneurysms and cardiac remodeling. Furthermore, the use of neutralizing antibodies against the leptin receptor has demonstrated improvements in cardiac function. These findings provide evidence supporting the role of endogenous leptin as a driver of cardiac hypertrophy [18,70]. This is possible through several pathways: rapamycin signaling, the activation of calcineurin and NFAT, the activation of MAPK 14 (p38), and increases in intracellular reactive oxygen species. However, providing adequate levels of leptin restores cardiac thickness to normal [18,119].

**Figure 2:**
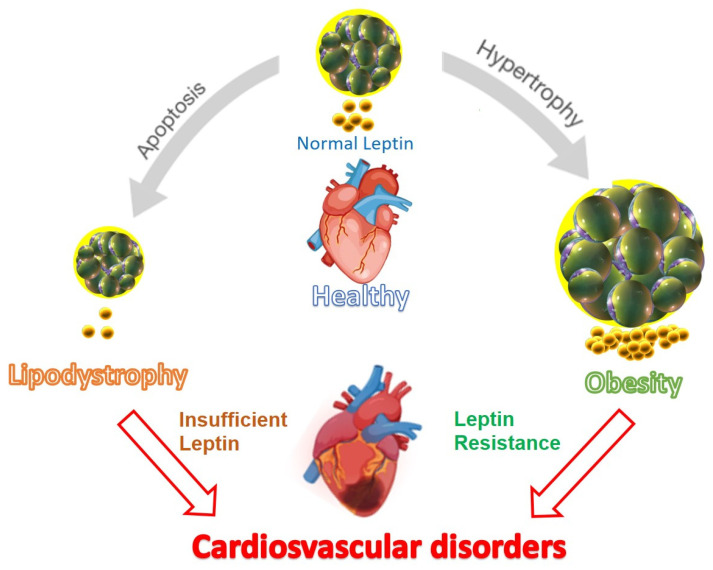
Paradoxical effect of leptin: relationship between circulating leptin levels and cardiovascular dysfunction. For proper cardiovascular function, it is essential to maintain circulating leptin levels within a narrow normal range. In conditions of lipodystrophy, resulting from widespread adipose tissue apoptosis or the inability to develop adipose tissue correctly, extremely low levels of circulating leptin promote cardiovascular disorder. Conversely, in diet-induced obesity, hyperleptinemia acts as an important factor in developing cardiovascular dysfunction due to leptin resistance (modified from reference [63]).

Hyperleptinemia has been observed in mice with diet-induced obesity, providing protection through the activation of the STAT3 pathway and beyond, compared to those with mutant LepR obesity [120]. Leptin has been shown to reduce myocardial reperfusion-induced death and the induced elongation of myocardial cells and eccentric dilation with compensation. On the other hand, the increase in heart rate and blood pressure mediated by leptin can lead to increased myocardial workload, contributing to long-term cardiac hypertrophy (through sympathetic nervous system activation) [68]. Therefore, there are contradictory results regarding whether leptin causes hypertrophy or, as noted in the study by Leifheit-Nestler et al. [121], is protective. Apparently, sex, patient nutritional status, leptin resistance, or age all influence this, so it is not clear whether obesity-induced cardiac hypertrophy is the result of leptin or resistance to its cardioprotective effects [18,22] (Figure 2).

Calcium entry into the heart serves as a multifunctional signal, influencing heart muscle contraction, regulating the duration of this action’s potential, and controlling gene expression. This ion plays a crucial role in heart excitation–contraction coupling, where β-adrenergic signals activate Na^+^/Ca^2+^ exchange channels through protein kinase A (PKA) signaling, resulting in sarcolemma depolarization. This depolarization opens L-type calcium channels, allowing calcium to enter the cytoplasm, initiating muscle contraction by triggering its release through ryanodine receptor channels in the sarcoplasmic reticulum. Sarcoplasmic/endoplasmic reticulum calcium ATPase 2 (SERCA2) and phospholamban are vital for calcium transport between myocytes and the cytoplasm. Disruptions in calcium circulation within the sarcoplasmic reticulum can lead to heart diseases such as heart failure and arteriosclerosis, contributing to disease progression. Understanding these mechanisms is crucial for comprehending the pathophysiology of cardiac disorders [122,123]. Studies conducted in mice with leptin deficiency revealed reduced activation of SERCA2 and Na^+^/Ca^2+^ channels. However, when leptin was administered to mice with ob gene mutations, there was an improvement in β-adrenergic signaling, increased PKA activation, and enhanced phospholamban phosphorylation. These findings suggest a correlation between leptin levels and cardiac function, emphasizing the role of leptin in influencing key molecular pathways involved in heart regulation. Further research in this area could provide valuable insights into the potential therapeutic implications of modulating leptin signaling for cardiac health [18,124].

The loss of cells in the heart due to myocardial apoptosis plays a crucial role in the progression of heart failure and compensatory remodeling in this disease [125]. TNF-α can induce apoptosis by binding to TNFR-1, contributing to CVD. In this scenario, treatment with leptin has been used, which interferes with the mitochondrial pathway, preventing apoptosis by TNF [126]. This suggests a potential role for leptin in mitigating apoptosis and its detrimental effects on the heart in the context of CVD.

In other words, leptin may play a protective role in stressful physiological situations [18], even though other authors have found that leptin may sensitize myocardiocytes to apoptosis [127]. In fact, disruption of leptin signaling leads to lipid accumulation and increased induction of cardiomyocyte apoptosis, which is resolved when adequate levels of leptin are reached [128]. Furthermore, in mice with cardiomyocyte-specific LepR deletions, this deficit in leptin signaling led to increased cardiac hypertrophy, apoptosis, impairment of cardiac structure and function, and impairment of energy, glucose, and fatty acid metabolism, further accelerating cardiac damage due to myocardial infarction [129].

Another effect of leptin that has been discovered is endothelium-dependent vasodilation through nitric oxide. In a state of leptin deficiency, vasodilation decreases, and arterial blood vessels contract more. However, this effect disappears when leptin levels are restored. The vasodilation induced by leptin can be explained by signaling [130,131,132]. Leptin triggers the phosphorylation of Akt, leading to the serine phosphorylation of eNOS, independently of PI3K. The latter produces nitric oxide (NO), which activates soluble guanylate cyclase and increases cyclic guanosine monophosphate synthesis in smooth muscle cells, ultimately resulting in vasodilation. Therefore, hyperleptinemia in obesity may act as a compensatory mechanism to overcome leptin resistance. While endothelial leptin signaling is considered protective against neointima formation in a healthy state, obesity-induced leptin resistance can shift this balance toward an atherogenic phenotype [68]. Consequently, hypertension and atherosclerosis can be triggered (due to a lack of dilation and continuous stimulation of the sympathetic nervous system). Hypertension is believed to result from the sympathetic system activation commonly found in obese patients, affecting peripheral resistance or blood flow in the kidneys, along with elevated leptin levels in these individuals [133]. In other words, increased plasma leptin levels, either through exogenous leptin administration or ectopic leptin overexpression, elevate blood pressure and heart rate, eventually leading to hypertension [134].

A novel mechanism of endothelial dysfunction and cardiac fibrosis in obesity is leptin-mediated aldosterone production, which impairs myocardial relaxation and contributes to CVD [135]. Therefore, spironolactone (an aldosterone antagonist) is used to reduce morbidity and mortality in obese patients with heart failure with a preserved ejection fraction [136].

Atherosclerosis is an indirect effect of leptin when its levels are very high [137]. It is mediated through the entry of monocytes into blood vessels, the transformation of macrophages into foam cells, the proliferation of vascular smooth muscle cells, and the secretion of atherosclerotic cytokines. Different studies have also reported that higher levels of leptin correlate with an increased number of stenotic coronary arteries and arterial stiffness in patients with coronary heart disease [68]. A previous study has shown the protective effects of leptin by reducing hepatic cholesterol (downregulating proinflammatory cytokines such as TNF-α or MCP-1) [138]. Moreover, leptin can activate AMPK, therefore inhibiting cholesterol synthesis [139]. This is not contradictory, as atherosclerotic effects occur when leptin levels are chronically elevated above their normal value and depend on the pathophysiological context of patients, medications, or other yet unidentified factors [18,68].

In other words, beneficial effects of leptin in CVD can be observed, but, in most cases, hyperleptinemia (especially when associated with obesity) exerts detrimental effects on CV function and promotes adverse outcomes in CV disorders, making it a significant marker for CV disorders in the obese population. This is referred to as the “paradoxical effect” [70]. 

Even in young patients with T1DM who are obese elevated levels of leptin have been observed, along with cardiovascular complications similar to those found in young patients with T2DM. Interestingly, the levels of other adipokines, such as adiponectin, were not observed to be as low as those seen in T2DM cases [140]. Consequently, it is plausible that leptin or leptin resistance may play a role in the cardiovascular complications associated with obesity and diabetes. 

Further evidence supporting the protective role of leptin in cardiovascular disease has been presented in studies involving patients with lipodystrophy [141]. Lipodystrophy is characterized by a partial or total absence of adipose tissue and very low levels of leptin, leading to severe metabolic derangements, including marked insulin resistance, type 2 diabetes, and cardiovascular disease (CVD). Indeed, treatment with leptin has been proposed as a potentially beneficial intervention for reducing the risk of CVD in these patients.

## 9. Leptin as a Therapeutic Target

Regarding obesity and obesity-associated DM, weight loss through restrictive diets or lifestyle modifications lead to a restoration of leptin sensitivity. This restoration could contribute to maintaining a certain body weight, preventing the development of obesity-associated comorbidities such as CVD [70,142].

Leptin-based therapy has demonstrated beneficial effects in obese patients with leptin gene mutations, restoring energy balance, glucose homeostasis, and neuroendocrine dysfunctions [12]. However, this therapy has shown limited effectiveness in common dietary obesity cases where hyperleptinemia already exists. To address leptin resistance, a combination of leptin and sensitizing therapies has been tested as a pharmacological strategy for weight loss. Recent studies have explored the inhibition of negative regulators of the leptin signaling pathway (SOCS3 and PTP1B) to enhance the effects of leptin administration in obese individuals. These inhibitors (such as thiazolidinediones and trodusquemine) suppress weight gain and reduce food intake and body weight in mice, as they cross the BBB, making them an interesting option to restore leptin responsiveness [143]. Moreover, altering leptin receptor endocytosis and intracellular transport is considered a possible approach to treating obesity (e.g., by fusing leptin with another molecule to improve its passage through the BBB, always confirming that this barrier is intact) [12,118,143].

In the context of leptin monotherapy, it is noted that most obese patients have elevated plasma leptin levels, making them unresponsive to these treatments. Sensitizers can be categorized into two groups: those that enhance the anorexigenic effect of exogenous leptin but have little effect on weight loss, such as meta-chlorophenyl piperazine, metformin, and betulinic acid [12], and compounds that induce weight loss in obese animals with hyperleptinemia and restore leptin sensitivity, such as glucagon-like peptide 1, cholecystokinin, and others [12].

Several of these sensitizers are used in DM treatment, as mentioned earlier, such as amylin, which enhances leptin action by increasing IL-6 in the hypothalamus and subsequently activating the STAT3 pathway [144,145]. Ozcan et al. [146] described celastrol as a significant leptin sensitizer (it inhibits 6-phosphofructoquinase in skeletal muscle and activates AMPK, leading to changes in glycolysis and fatty acid oxidation in muscle), in addition to suppressing food intake, increasing energy expenditure, and reducing body weight in patients with elevated leptin levels. This mechanism is still under study, but it is assumed to be related to the expression of IL-1 receptor 1 in the hypothalamus [147,148,149].

Leptin analogs, such as metreleptin, are employed in the treatment of congenital leptin deficiency and/or obesity. Metreleptin, which was approved in Japan in 2013, shares similar functions with endogenous leptin. It aids in reducing body mass and food intake while suppressing hunger. Additionally, this analog seems to lower plasma glucose levels and enhance insulin sensitivity, suggesting potential applications in T2DM [150].

Maintaining well-controlled circulating leptin levels is crucial for proper cardiac function. Both leptin deficiency and hyperleptinemia, which is more common in diet-induced obesity, are associated with cardiovascular disorders and increased mortality rates [151]. Therefore, understanding and regulating leptin levels are of interest in the context of cardiovascular pathology [21].

## 10. Conclusions

Given the anticipated growth rates of obesity in the coming decades, further research into the functions and mechanisms of specific adipokines, such as leptin, is crucial. This exploration could pave the way for more targeted therapeutic strategies. Individuals with obesity, especially those at high risk of CVD, may benefit from intensified CVD prevention interventions.

In the absence of specific targeted therapies, it is imperative to pay special attention to these high-risk individuals. They should be educated about the potential consequences of poorly managed T2DM or a high BMI. Lifestyle modifications, which also influence leptin levels, should be encouraged as a proactive measure. Additionally, considering some of the antidiabetic drugs mentioned earlier may offer further avenues for managing the associated risks.

Concerning leptin, a pivotal appetite suppressant, there are currently no therapeutic strategies utilizing it for obese individuals, except in cases where it is employed as replacement therapy for patients with inactivating mutations in the leptin gene. This restricted application may arise from the fact that disrupted leptin and receptor expression frequently result in leptin resistance, a crucial factor in the development of complications associated with obesity.

Hence, it would be intriguing to investigate potential drugs that can reverse this resistance, enabling leptin to effectively carry out its function in the hypothalamus. This reversal could help in reducing food intake, providing a sense of fullness, and ultimately preventing associated complications, such as CVD.

Understanding the role of leptin in CVD is indeed complex, as it has been associated with both protective and risk factors. Leptin has demonstrated effects that promote inflammation, thrombosis, arteriosclerosis, and angiogenesis. However, the interplay between leptin and adiponectin, another adipokine, is crucial in influencing the progression of cardiovascular disease.

To gain a comprehensive understanding, it is pertinent to investigate potential alterations in the signaling pathways of leptin and adiponectin in individuals with cardiovascular conditions. Examining how these adipokines interact and impact key molecular pathways could provide valuable insights into their roles in cardiovascular health and potentially lead to targeted interventions for CVD management. Enhancing leptin sensitivity may potentially amplify the positive metabolic effects and beneficial cardiac actions of leptin while mitigating its proinflammatory impact. Subsequent investigations are warranted to validate this hypothesis.

Nevertheless, the precise relationship between hyperleptinemia and CVD remains incompletely defined. It is still uncertain whether hyperleptinemia itself or the existence of leptin resistance in obese individuals contributes to CVD. The conflicting findings in these instances suggest that additional factors, such as age, sex, BMI, and dietary habits, should be considered when evaluating the influence of leptin on cardiovascular health.
